# Role of Lung Ultrasonography in Acute Respiratory Distress in Pediatric Age Group: A Prospective Single-Centre Study

**DOI:** 10.7759/cureus.61385

**Published:** 2024-05-30

**Authors:** Aishwarya Jeyakumar, Venkata Sai Pulivadula Mohanarangam, Vignesh Gadupudi

**Affiliations:** 1 Radiology, Sri Ramachandra Institute of Higher Education and Research, Chennai, IND; 2 Radiodiagnosis, Sri Ramachandra Institute of Higher Education and Research, Chennai, IND

**Keywords:** bronchiolitis, walri, pneumonia, chest x rays, lung ultrasound

## Abstract

Introduction

Lung diseases are the most frequently encountered form of diseases primarily affecting infants under one year of age. Although the chest X-ray is the first modality of choice, ultrasonography (USG) has emerged as an alternative. Lung ultrasound (LUS) finds its application in the evaluation of several pediatric lung diseases.

Objective

To assess the use of LUS in acute lower respiratory infections and assess the correlation between etiological diagnosis and radiological diagnosis.

Methods

This was a hospital-based prospective observational study conducted with children presenting with upper respiratory infections. Around 97 children were included in the study. Clinical diagnosis was made by the pediatrician. LUS was performed by a trained radiologist, using the two-dimensional (2D) ultrasound mode and motion mode (M mode) to assess the LUS in the respective areas of the chest, thereby assessing bilateral lung fields for these patients.

Results

The majority of our study participants were under one year old (87%), and more than half were male (55%). Bronchiolitis and lower respiratory tract infections (LRIs) were the most commonly seen clinical diagnoses. The distribution of USG findings was statistically significant across the clinical diagnosis (p-value < 0.05).

Conclusion

Our study found that LUS can serve as an important tool for diagnosing several acute respiratory diseases. It also showed that LUS can replace X-rays in cases of children diagnosed with acute respiratory diseases.

## Introduction

Lung diseases are the most frequently encountered form of disease primarily affecting infants under one year of age. These still cause high rates of mortality and morbidity among this age group, and they are the primary causes of death among children under five [1]. Therefore, the current focus is on several investigatory modalities, alongside clinical examination, to assist us in reaching a timely and accurate diagnosis of several critical illnesses which are hard to diagnose, thereby enabling early treatment and ensuring better prognosis [2].

Chest X-rays and CT remain the main investigation modalities of choice for diagnosing most lung diseases among the pediatric population [3]. It has recently been observed that chest X-rays have their own drawbacks, despite the invention of bedside X-rays, as bedside X-rays are often not as efficient as the conventional ones. Furthermore, conventional X-rays require cases to be transferred to the X-ray rooms, which increases the risk of infections and other disease transmissions, in addition to exposure to radiation. CT scans also possess similar drawbacks with higher exposure to radiation, compared with X-rays [4].

Ultrasonography (USG) has emerged as an alternative to other imaging modalities in diagnosing several severe illnesses. It was long thought that USG and its utilization in the diagnosis of respiratory illnesses is due to its inability to pass through gas-filled respiratory structures. Recently, advances in knowledge on USG have led to the discovery that ultrasound produces artifacts when passed through abnormal tissue interfaces, hollow surfaces, or gas-filled structures. Thus, USG has been widely identified as an important modality of investigation in diagnosing several respiratory illnesses and is mainly based on the scatter and reflection of ultrasound beams by different media [5].

Lung ultrasound (LUS) now finds its application in the evaluation of several pediatric lung diseases for various reasons, such as less subcutaneous fat in the pediatric chest wall. The partially ossified nature of the pediatric chest wall also tends to provide an additional acoustic medium that is generally not seen among adults. In addition, the presence of the thymus gland also enhances the acoustic window for the visualization of the mediastinum and anterior chest wall among smaller children. Furthermore, the non-ossified costal and sternal cartilages that are usually hypoechoic also add to the advantages of using ultrasound for the pediatric age group population [6].

The common indications for which LUS finds its application include evaluating the opacities and other pleural abnormalities detected through X-rays, especially differentiating if the opacities are caused by pleural or parenchymal diseases, thereby aiding in prompt management of cases [7]. Another important application of LUS among pediatric children is in the evaluation of mediastinal widening and in further investigating chest wall diseases. LUS also enables the radiologist in determining if the lesion is purely cystic or solid [8].

Despite these advantages, the use of LUS in the pediatric population has the following drawbacks: i) lack of supportive evidence; ii) the importance of finding an appropriate acoustic window; iii) contact between the affected lung portion and pleura; iv) difficulty in converting the acoustic phenomenon into images; and v) difficulty in differentiating consolidation and atelectasis. Despite the use of LUS in detecting respiratory illnesses among the pediatric population, few studies have explored its application in India. In addition, studies exploring the use of LUS compared with X-rays are also rare, especially in a South Indian setting.

The main aim of the study is to assess the following: the role of LUS in acute lower respiratory infection patterns of pediatric patients in the age groups of one month to five years, the correlation between clinical diagnosis and radiological diagnosis, and the correlation between the findings of chest X-rays and those of LUS. 

## Materials and methods

A hospital-based prospective observational study was conducted in the radiology department of the Sri Ramachandra Institute of Higher Education and Research, Chennai, from November 2020 to November 2022. As part of this research proposal, we intended to assess the use of LUS in acute lower respiratory infections and to assess the correlation between clinical/etiological diagnosis and radiological diagnosis. After Institutional Ethics Committee approval and informed written consent was obtained, around 97 children with lower respiratory infections were selected based on the inclusion and exclusion criteria. The inclusion criteria were pediatric patients with complaints of fever, tachypnea, cough, wheezing, cold, running nose, and those between the ages of one month to five years. Excluded from the study were any pediatric patients with known cases of cardiogenic edema/congenital airway malformation/ bronchopulmonary dysplasia/chronic lung diseases/hemodynamic instability and children over the age of five years.

Study procedure

Institutional Ethics Committee clearance was obtained. All the children were assessed for the above-mentioned inclusion and exclusion criteria. The parents of those patients who fulfilled the criteria were approached for informed written assent. A sample size of 97 patients were enrolled in the study. After the children’s parents or their relatives/bystanders were provided with an explanation regarding the study, consent was taken. Basic sociodemographic details of the patients were obtained using a semi-structured proforma. The patients were referred by the pediatrician to radiologists who have trained in LUS. In order to assess the disease, we used stationary/portable ultrasound machines to evaluate lung pathologies that can be diagnosed by LUS. Two types of probes were used: a 6-cm convex probe with a frequency ranging from 1 to 6 MHz and a 6-cm linear probe with a frequency ranging from 5 to 9 MHz. The probes were kept in the designated areas, as mentioned below, and the LUS results were recorded in the standard format. We recorded both brightness mode (B mode) and motion mode (M mode) findings in LUS. LUS is a rapidly growing field of study, as the technique is considered to be cost-effective, radiation-free, and a readily available alternative to standard conventional X-ray and CT imaging.

Assessment areas, positions, and modes

Each hemithorax was divided into anterior, lateral, and posterior zones/regions by anterior and posterior axillary lines. Each zone/region was further divided into upper and lower regions. The dorsal region of the upper lobes was not easy to assess as the scapula does not allow an acoustic window. The patient was examined in a supine/semi-recumbent position with the arm abducted to facilitate examination of the anterior and lateral chest wall. A lateral decubitus position or minimal tilting with upper limb moved anteriorly was required to expose the lateral and posterior chest wall for examination. The dorsal region of the lower lobes was better appreciated in the lateral decubitus position. 

Statistical methods

Data were entered in Excel and analyzed using SPSS version 20, and graphs were created using Microsoft Excel/SPSS. Continuous variables such as age were summarized as mean ± SD or median with an interquartile range based on normality. The percentage of individuals with a clinical diagnosis was summarized as frequency and proportions. The association between the clinical diagnosis and other LUS findings (B mode) was assessed using the chi-square test or Fisher’s exact test. The association between the LUS findings (B mode) and the X-ray findings was assessed using the chi-square test or Fisher’s exact test. A p-value of < 0.05 was taken to be statistically significant.

## Results

97 patients that met the inclusion criteria were recruited. Since all the patients parent's consented to take part, the study had a 100% response rate. LUS was used for all patients with upper or lower respiratory tract symptoms. Next, an X-ray investigation was performed on patients suspecting the lower respiratory tract infection (LRI). Finally, we contrasted the results of the two experiments.

**Figure 1:**
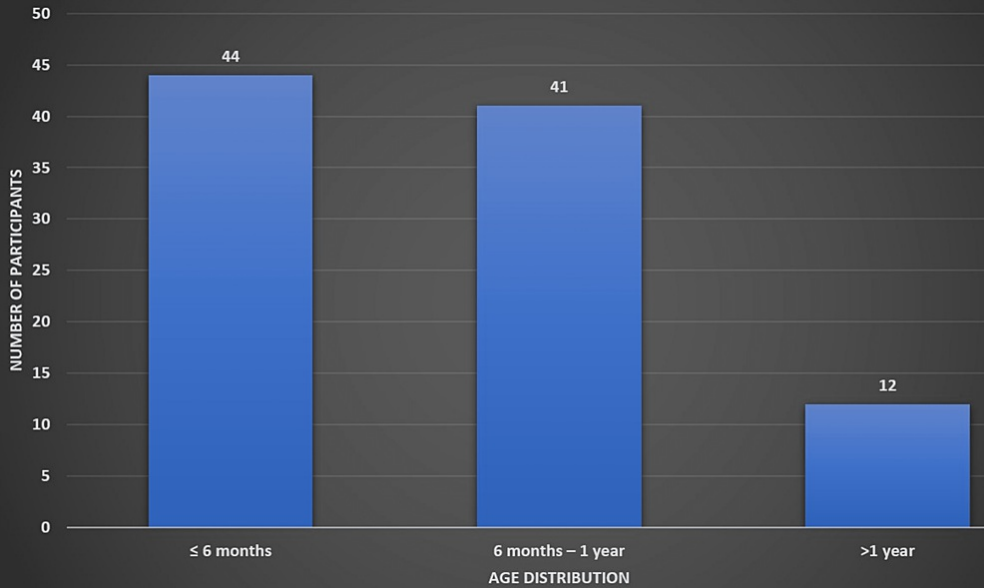
Age distribution among the study participants Figure created by Vignesh Gadupudi

Nearly four out of every five (80%) of the study's participants were infants under the age of one year. Figure 1 shows the age distribution among the study participants.

**Figure 2:**
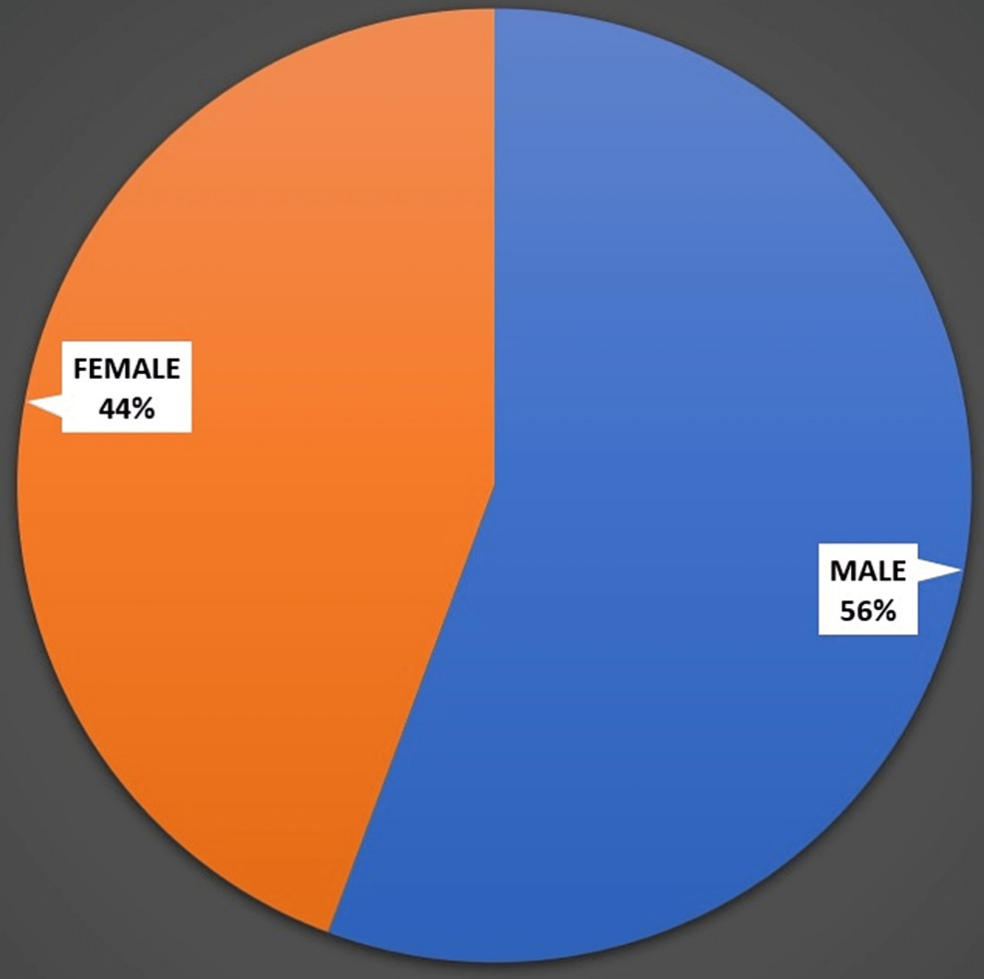
Gender distribution among the study participants Figure created by Vignesh Gadupudi

Boys made up 56% of the population, while females made up 44%. Figure 2 shows the gender distribution among the study participants. 

**Figure 3:**
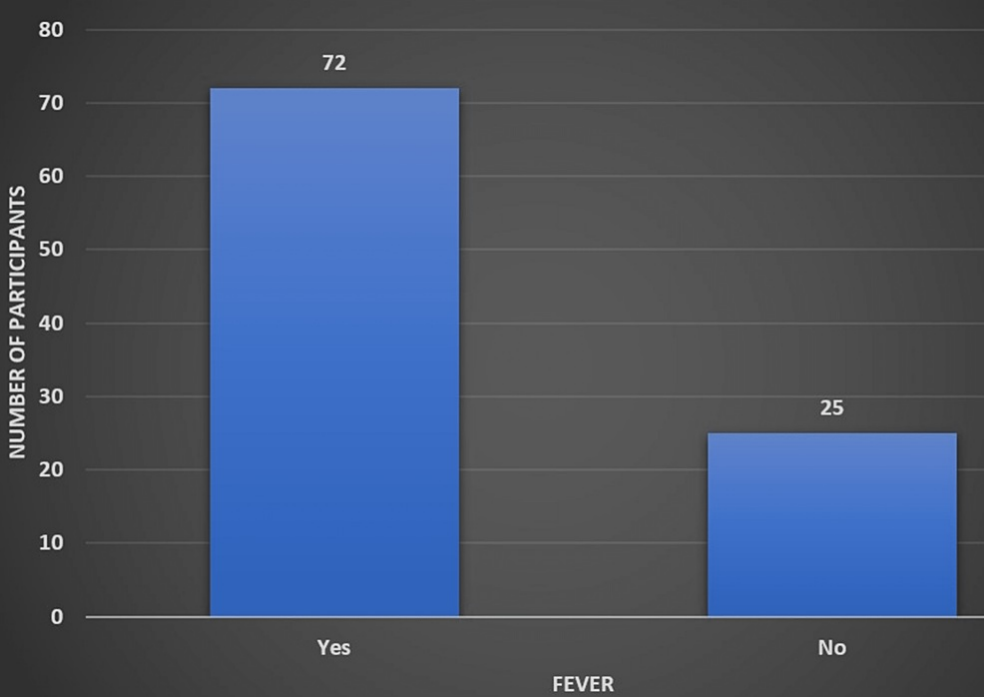
Distribution of fever among the study participants Figure created by Vignesh Gadupudi

Fever was the presenting symptom in nearly three-quarters (75%) of the patients. Figure 3 shows the distribution of fever among the study participants.

**Figure 4:**
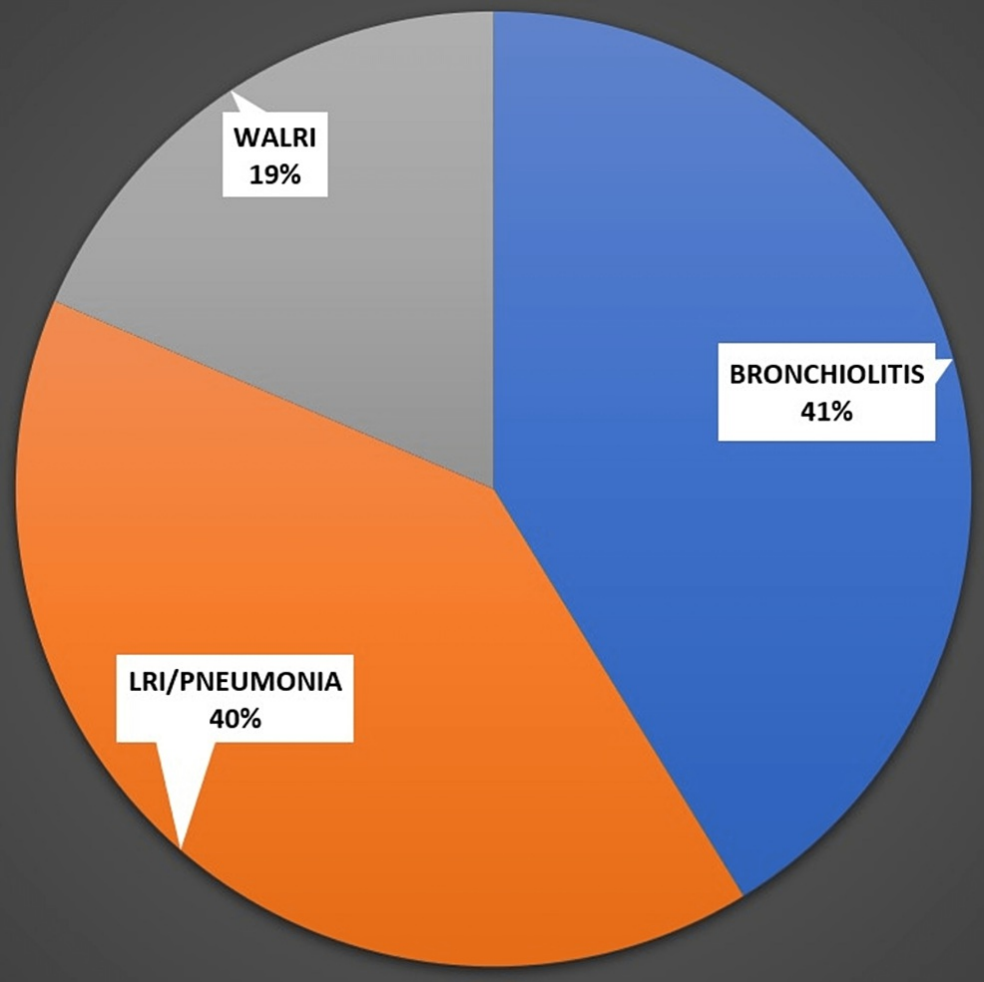
Clinical diagnosis among the study participants LRI: Lower respiratory tract infection; WALRI: Wheeze-associated lower respiratory tract infection

LRI and bronchiolitis were the most frequently seen diagnosis accounting for nearly 40% each. Figure 4 shows the clinical diagnosis among the study participants.

USG identified more abnormal cases in comparison to X-rays. The distribution of USG findings was statistically significant with respect to X-ray findings (p-value < 0.05). Table 1 explains the association between USG findings (left side) and X-ray findings (left side) among the study participants.

**Table 1:** Comparison between USG findings (left side) and X-ray findings (left side) among the study participants (N=97) USG: Ultrasonography

Characteristics	X-ray (Abnormal)	X-ray (Normal)	P-value
USG			
Abnormal	15 (26.3)	42 (73.7)	0.05
Normal	0 (0.0)	10 (100)	

The distribution of USG findings among the lung fields was found to be statistically significant from the clinical diagnosis observed (p-value < 0.05). All diagnoses were found to be affecting bilateral lung fields in the USG examination. Table 2 explains the association between the clinical diagnosis and USG findings among the study participants.

**Table 2:** Association between the clinical diagnosis and USG findings among the study participants (N=97) LRI: Lower respiratory tract infection; WALRI: Wheeze-associated lower respiratory tract infection; USG: Ultrasonography

	Right lung affected	Left lung affected	Both lungs affected	P value
Bronchiolitis	3 (7.5)	6 (15.0)	31 (77.5)	0.03
LRI	6 (15.4)	0 (0.0)	33 (84.6)	
WALRI	5 (27.7)	2 (11.1)	11 (61.1)	

The distribution of USG findings was statistically significant with the clinical diagnosis, where A lines were the common findings in the case of bronchiolitis, consolidation in the case of LRI, and B lines in the case of wheeze-associated lower respiratory tract infection (WALRI). Table 3 explains the association between the USG findings in either lung with clinical diagnosis.

**Table 3:** Association between USG findings in either lung with clinical diagnosis among the study participants (N=97) LRI: Lower respiratory tract infection; WALRI: Wheeze-associated lower respiratory tract infection; USG: Ultrasonography

	Bronchioliitis	LRI	WALRI	P-value
A line	20 (50.0)	1 (2.5)	2 (11.1)	0.04
B lines	6 (15.0)	3 (7.6)	2 (11.1)	
B lines (2-3)	7 (17.5)	4 (10.2)	7 (38.8)	
B rockets	3 (7.5)	6 (15.3)	4 (22.2)	
Consolidation	4 (10.0)	25 (64.1)	3 (16.6)	

Representative cases 

Case 1: Wheeze-associated lower respiratory tract infection

**Figure 5:**
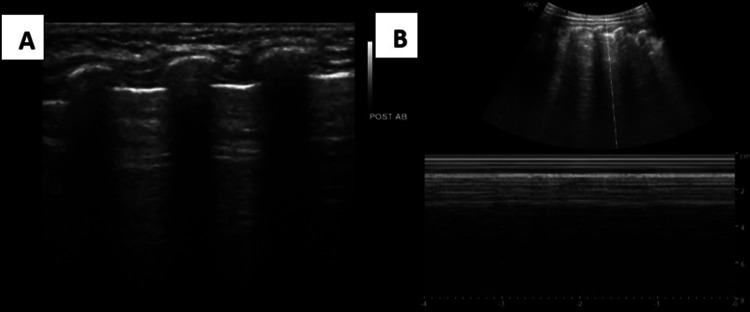
A) B-mode ultrasound in the left posterior upper region shows normal pleural line and A-lines; B) M-mode ultrasound showing “Sand on the Beach” appearance. B mode: Brightness mode; M mode: Motion mode

B-Mode ultrasound in the left posterior upper and lower regions shows normal pleural line and A-lines and M-mode ultrasound show a “sand on the beach” appearance (Figure 5).

Case 2: Consolidation (Lower Respiratory Tract Infection)

**Figure 6:**
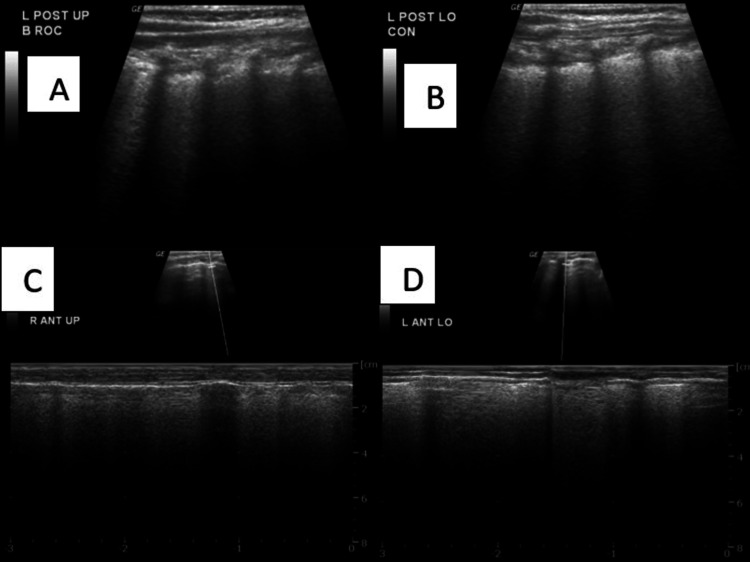
A) B-mode ultrasound in the left posterior upper region shows a thick irregular pleural line combined with a thick vertical hyperechoic stripe (C- line); B) B-mode ultrasound in the left posterior lower region shows a thick irregular pleural line combined with a thick vertical hyperechoic stripe (C- line); C,D) M-mode ultrasound confirming the presence of the C line. B mode: Brightness mode; M mode: Motion mode

B-mode ultrasound in the left posterior upper and lower regions shows a thick irregular pleural line combined with a thick vertical hyperechoic stripe (C-line) which indicates the presence of consolidation and M-mode ultrasound confirms the presence of the C-line (Figure 6).

Case 3: Bronchiolitis

**Figure 7:**
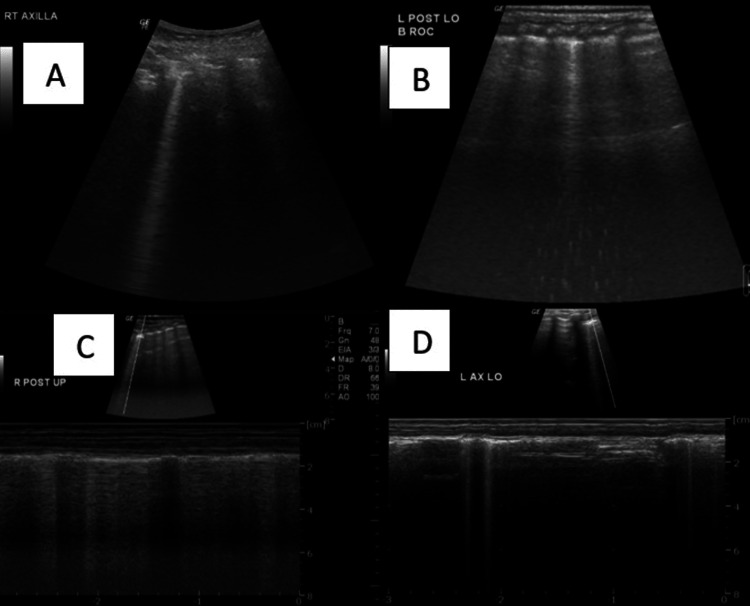
A) B-mode ultrasound in the right axillary region shows a single B line; B) B-mode ultrasound in the left posterior lower region shows a single B line; C,D) M-mode ultrasound confirming the presence of the B line. B mode: Brightness mode; M mode: Motion mode

B-mode ultrasound in the right axillary region and left posterior lower regions show a single B line and M-mode ultrasound confirms the presence of the B line (Figure 7).

**Figure 8:**
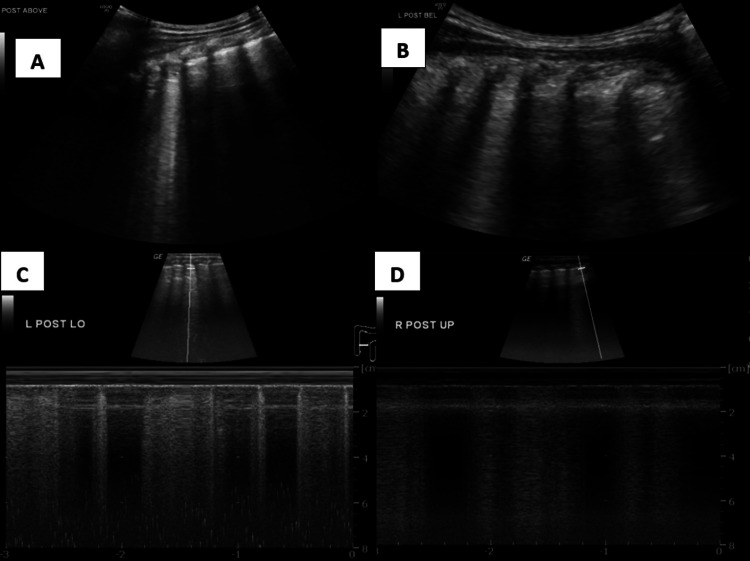
A) B-mode ultrasound in the posterior upper region showing hyperechoic lung rockets; B) B-mode ultrasound in the posterior lower region showing hyperechoic lung rockets; C,D) M mode confirming the presence of lung rockets. B mode: Brightness mode; M mode: Motion mode

B-mode ultrasound in the posterior upper and lower regions showed hyperechoic lung rockets and M-mode confirmed the presence of lung rockets (Figure 8).

## Discussion

USG has emerged as an alternative to other imaging modalities in diagnosing several severe illnesses. It was long thought that USG and its utilization in the diagnosis of respiratory illnesses is due to its inability to pass through gas-filled respiratory structures [9]. Recently, advances in knowledge on USG have led to the discovery that ultrasound produces artifacts when passing through abnormal tissue interfaces, hollow surfaces, or gas-filled structures, which can be used to our advantage.

LUS is used in the diagnosis of several respiratory diseases such as pleural abnormalities, bacterial and viral infections, bronchiolitis, and LRIs. LUS has an added advantage in that it is devoid of ionizing radiations and can be done as a bedside test for critically ill children. Several studies have tried to establish the importance of LUS in detecting respiratory lesions in cases of children suffering from pulmonary symptoms [10]. Our study was one of the few studies that aimed at assessing the use of LUS in diagnosing acute lower respiratory infections and at establishing the correlation between etiological diagnosis and radiological diagnosis, using X-rays and lung USG.

In our study, we observed that the majority of the participants belonged to the age group of under one-year-old, with a mean age of 10.4 (6.7) months. The majority were found to be males (56%). We observed that fever was the most common symptom observed among the children who presented with respiratory symptoms. The most common respiratory diagnoses made by the pediatrician were bronchiolitis and pneumonia. This was in line with findings from other study settings [11,12]. 

Of the various regions scanned under B mode using the LUS, we found that the pattern of USG findings was significantly different across the clinical diagnosis in the right upper posterior region (p-value 0.04). The other regions did not show any statistically significant difference. When the X-ray findings were compared with the USG findings, we observed that the distribution of USG findings was statistically significant compared with the X-ray findings (p-value < 0.05). USG detected more abnormal findings on the left side compared with the X-rays. The finding that USG detects a higher number of cases with respiratory symptoms than X-rays was documented in previous studies [13,14]. We did not observe a statistically significant difference when we compared X-ray and USG findings on the right side.

We compared the association between chest X-ray findings and USG findings, during which we observed that the distribution of USG findings among the right lung, left lung, and both lungs affected was significantly different across the clinical diagnosis observed (p-value < 0.05). All diagnoses were affecting both the lung fields in USG, which shows the importance of USG in diagnosing and detecting more cases with respiratory symptoms. Several previous studies also established that USG is a better investigatory modality to detect respiratory infections which present with subtle clinical symptoms [15,16].

We tried to establish an association between USG findings in either lung with the clinical diagnosis. We observed that the distribution of USG findings was statistically significant across the clinical diagnosis, where A lines were the common findings in cases of bronchiolitis, consolidation in cases of LRI, and B lines in cases of WALRI. Such a distribution of USG patterns was also on par with previous findings in the literature [17].

Our study findings have demonstrated that LUS can serve as an important tool in the diagnosis and prognostication of children with respiratory illnesses. LUS can substantially augment the other treatment modalities that are available, with an added advantage being that it is easy and quick to perform, without any side effects of exposure to ionizing radiation. LUS can serve as a multi-faceted tool that can be adapted by various clinical practitioners such as intensivists, anesthesiologists, pediatricians, pulmonologists, radiologists, and emergency physicians. Many studies have shown that the results obtained from LUS are on par with the results from CT scans and are even better than X-rays. A recent study by Amatya et al. with 62 patients estimated that LUS demonstrated a sensitivity of about 91% compared with X-rays (73%) in diagnosing respiratory infections, with CT as the gold standard (p < 0.01) [18].

A meta-analysis of five studies by Ye et al. compared the ability of LUS to detect respiratory cases compared with X-rays, using CT as the gold standard. Ye et al. found that the pooled sensitivity estimate of LUS was found to be 95% (93%-97%) and the specificity was 90% (86%-94%) compared with X-rays, which had poorer sensitivity and specificity of 77% and 90% respectively in detecting lesions among children with respiratory symptoms [19]. LUS is also a useful tool to complement cardiac windows. The main limitation is that different experts might provide different interpretations for the lesions in LUS, thus leading to lower interobserver agreement.

Future implications

LUS could help clinicians in differentiating viral and bacterial pneumonia, and in diagnosing interstitial pneumonia. It could also help clinicians to start treatment early and help in prognostication. It is easy to use and can be utilized even in resource-poor settings. As several artifacts can be interpreted in different ways by different clinicians, training clinicians is necessary to increase interobserver agreement. The development of artificial intelligence techniques can also augment the findings of LUS in the near future.

Strengths

Our study was one among very few studies that have estimated the usefulness of LUS in diagnosing acute respiratory infections in a South Indian setting. We compared the findings obtained with LUS with the X-ray findings to establish the superiority of LUS over X-ray. We also established the various LUS findings and patterns observed in common acute respiratory conditions so the children such as WALRI/bronchiolitis and pneumonia.

 Limitations

The main limitation was a small sample size to evaluate the diagnostic accuracy of LUS. We did not compare the findings of LUS with a gold-standard CT to estimate its diagnostic ability. The study was conducted at one single center in South India, so the findings are only generalizable to similar study settings. The findings of LUS and X-rays were determined by radiologists and clinicians; thus, we could not omit the subjective component which would have led to subjective bias, interviewer bias, and interobserver agreement.

## Conclusions

Our study found that LUS can serve as an important tool for diagnosing several respiratory diseases. It can serve as the first line of investigation and also as a substitute for X-rays to diagnose pneumonia/WALRI/bronchiolitis in children, as it detected more subtle lesions without the side effects of exposure to ionizing radiation. Furthermore, LUS can serve as a dynamic diagnostic tool that can perform real-time imaging of the movement of organs that is synchronized with the respiratory cycle.

## References

[1] Harris M, Clark J, Coote N, Fletcher P, Harnden A, McKean M, Thomson A (2011). British Thoracic Society guidelines for the management of community acquired pneumonia in children: update 2011. Thorax.

[2] Chiumello D, Sferrazza Papa GF, Artigas A (2019). ERS statement on chest imaging in acute respiratory failure. Eur Respir J.

[3] Lucaya J. Strife JL (2008). Pediatric chest imaging: chest Imaging in Infants and Children. Pediatric Radiology, 39(Suppl.

[4] Biagi C, Pierantoni L, Baldazzi M (2018). Lung ultrasound for the diagnosis of pneumonia in children with acute bronchiolitis. BMC Pulm Med.

[5] Berant R, Kwan C, Fischer J (2015). Emergency point-of-care ultrasound assessment of whiteout lung in the pediatric emergency department. Pediatr Emerg Care.

[6] Boursiani C, Tsolia M, Koumanidou C (2017). Lung ultrasound as first-line examination for the diagnosis of community-acquired pneumonia in children. Pediatr Emerg Care.

[7] Fuhlbrigge AL, Choi AM Weinberger SE, Drazen JM (2005). Diagnostic procedures in respiratory diseases. Harrison’s Principles of Internal Medicine.

[8] Desai SR, Hansell DM (1997). Lung imaging in the adult respiratory distress syndrome: current practice and new insights. Intensive Care Med.

[9] Musolino AM, Tomà P, De Rose C (2021). Ten years of pediatric lung ultrasound: a narrative review. Front Physiol.

[10] Caiulo VA, Gargani L, Caiulo S (2013). Lung ultrasound characteristics of community-acquired pneumonia in hospitalized children. Pediatr Pulmonol.

[11] Parikh K, Hall M, Mittal V (2014). Establishing benchmarks for the hospitalized care of children with asthma, bronchiolitis, and pneumonia. Pediatrics.

[12] Willson DF, Landrigan CP, Horn SD, Smout RJ (2003). Complications in infants hospitalized for bronchiolitis or respiratory syncytial virus pneumonia. J Pediatr.

[13] El-Malah HE, Hany S, Mahmoud MK, Ali AM (2015). Lung ultrasonography in evaluation of neonatal respiratory distress syndrome. Egyptian J Radiol Nucl Med.

[14] Toro MS, Martínez JL, Falcão RV, Prata-Barbosa A, Cunha AJ (2021). Point-of-care ultrasound by the pediatrician in the diagnosis and follow-up of community-acquired pneumonia. J Pediatr (Rio J).

[15] Magrelli S, Valentini P, De Rose C, Morello R, Buonsenso D (2021). Classification of lung disease in children by using lung ultrasound images and deep convolutional neural network. Front Physiol.

[16] Chavez MA, Shams N, Ellington LE (2014). Lung ultrasound for the diagnosis of pneumonia in adults: a systematic review and meta-analysis. Respir Res.

[17] Silva S, Biendel C, Ruiz J (2013). Usefulness of cardiothoracic chest ultrasound in the management of acute respiratory failure in critical care practice. Chest.

[18] Amatya Y, Rupp J, Russell FM, Saunders J, Bales B, House DR (2018). Diagnostic use of lung ultrasound compared to chest radiograph for suspected pneumonia in a resource-limited setting. Int J Emerg Med.

[19] Ye X, Xiao H, Chen B, Zhang S (2015). Accuracy of lung ultrasonography versus chest radiography for the diagnosis of adult community-acquired pneumonia: review of the literature and meta-analysis. PLoS One.

